# Knowledge, Perceptions, and Attitudes Regarding Psoriasis Among the General Population in Jeddah, Saudi Arabia

**DOI:** 10.7759/cureus.107102

**Published:** 2026-04-15

**Authors:** Tala Roblah, Leena Alotaibi, Mehad Almoqati, Majda Al-Sharif, Jehad Hariri, Mohammed Abduljabbar

**Affiliations:** 1 General Medicine, East Jeddah General Hospital, Jeddah, SAU; 2 General Medicine, King Abdulaziz Hospital, Makkah, SAU; 3 College of Medicine, King Abdulaziz University Faculty of Medicine, Jeddah, SAU; 4 Department of Dermatology, King Abdulaziz University Faculty of Medicine, Jeddah, SAU

**Keywords:** attitude, awareness, knowledge, population, psoriasis

## Abstract

Background and objective

Psoriasis is a chronic, immune-mediated inflammatory disease associated with a significant psychosocial burden and stigma. Misconceptions regarding its contagiousness and causes may negatively affect patients’ social interactions and quality of life. This study aimed to assess the knowledge, perceptions, and attitudes regarding psoriasis among the general population in Jeddah, Saudi Arabia.

Methods

We carried out a cross-sectional study with 526 participants, and used a snowball sampling method for recruitment. Participants were recruited through social media platforms, particularly WhatsApp, and received detailed information about the study before providing digital consent to participate. We used a questionnaire comprising four sections to collect data on sociodemographic data, awareness of psoriasis, knowledge assessment, and attitude evaluation.

Results

Overall, 89.5% of participants reported that they had heard or read about psoriasis, primarily through social media (28.1%) and through relatives or friends (27%). Knowledge levels varied, with 39.7% demonstrating good knowledge and 20.3% demonstrating poor knowledge. Higher education was significantly associated with better knowledge (p = 0.029), whereas other demographic factors showed no significant association. Regarding attitudes, 60.3% of the participants expressed positive perceptions toward psoriasis patients, whereas 27% expressed negative attitudes. Positive attitudes were significantly associated with obtaining information from books or lectures or through personal experiences with the disease (p = 0.042) and having a higher knowledge level (p < 0.001). We found no significant associations between attitudes and demographic factors. These findings suggest the need for targeted educational initiatives to improve public knowledge and attitudes toward psoriasis.

Conclusions

This study assessed the level of knowledge regarding psoriasis among the general population in the western region of Saudi Arabia. The results revealed the presence of negative attitudes toward patients with psoriasis. Thus, awareness of psoriasis in the general population needs to be improved.

## Introduction

Psoriasis is a common chronic inflammatory illness caused by the immune system and affects the skin, nails, and joints of people of all ages, including children and adults [1]. It is characterized by chronic inflammation leading to uncontrolled keratinocyte proliferation and impaired differentiation [2]. Genetic factors play an important role in the pathophysiology of psoriasis, and Psoriasis Susceptibility 1 (*PSORS1*) has been identified as a key genetic determinant. This gene is located within a 220 kb region of the major histocompatibility complex on chromosome 6p21 [3]. It is associated with the early onset of the disease, marked by increased severity and instability [4]. Plaque psoriasis is the most common form of psoriasis. Significant progress has been made in understanding the underlying causes, genetic predispositions, associated comorbidities, and development of biological therapies. This condition is frequently associated with psoriatic arthritis, cardiovascular and metabolic disorders, and depression [5]. The global prevalence of psoriasis is approximately 2%; however, this figure varies by country [6].

The prevalence of psoriasis is lower in Asian and certain African populations, while it can reach up to 11% in Caucasian and Scandinavian populations [7]. The prevalence of psoriasis in Saudi Arabia ranges between 2.47% and 5.3%, with the condition most commonly presenting before the age of 30 years. Moreover, the northern region, notably Al Jouf, has the highest documented rates [8,9]. Psoriasis care requires a multifaceted approach that can be improved with timely intervention, professional dermatological consultation, patient education about the disease and its medications, proactive control of risk factors, comorbidities, and disease progression [10]. The prevalence of psoriasis varies widely across populations and ethnic groups. Adult prevalence estimates range from 0.51% to 11.43%, whereas the prevalence among children ranges from 0% to 1.37% [11].

Relatives of patients with psoriasis are more likely to develop the disorder than individuals in the general population, implying that genetic factors may play a role in the pathogenesis of this condition [12]. Furthermore, psoriasis can affect many aspects of patients' lives, making it challenging to assess its social burden, particularly with regard to its effects on interpersonal relationships [13]. Misconceptions about psoriasis as a contagious disorder contribute to patient stigma and social exclusion, leading to marginalization in settings such as schools, workplaces, and swimming pools, which can negatively influence the social lives of patients with psoriasis [14]. According to previous research, depressive symptoms are more severe in individuals who are stigmatized in social contexts than in those who are not [15,16]. This study aimed to assess knowledge, perceptions, and attitudes regarding psoriasis among the general population in Jeddah, Saudi Arabia.

## Materials and methods

Study design and setting

This cross-sectional study was conducted among the general population in Jeddah, Saudi Arabia, from September to November 2024, using an online questionnaire distributed via social media platforms

Study participants

The inclusion criteria were residents of Jeddah, Saudi Arabia, aged ≥ 18 years, of both genders. The exclusion criteria were those who declined participation or those providing incomplete responses.

Sample size and sampling technique 

Snowball sampling was used to recruit potential participants. After applying the exclusion criteria, participants who met the inclusion criteria and agreed to join the study completed the questionnaire. The total sample was 526 participants.

Data collection

We used a questionnaire to collect data from a previous Saudi study. Participants were recruited via social media and WhatsApp and received a link to the online questionnaire, along with detailed information about the study's objectives, before participation. Digital informed consent was obtained through the questionnaire. Initially, the questionnaire was developed in English and carefully translated into Arabic by two bilingual translators to ensure accuracy and cultural relevance through collaborative cross-checking and resolution of discrepancies.

The first section of the questionnaire included sociodemographic characteristics, such as age, gender, marital status, education level, and occupation. The second section was designed to assess whether participants had heard or read about psoriasis and, if so, to identify their source of information. The third section of the survey included three closed-ended questions to assess the participants’ knowledge regarding psoriasis: “Can specific types of food exacerbate psoriasis? Are patients with eczema susceptible to psoriasis?” and “Is psoriasis an infectious disease?” The fourth section assessed the participants’ attitudes toward patients with psoriasis.

For knowledge questions, a score of “1” was assigned to correct answers, whereas a score of “0” was assigned to incorrect answers. Regarding attitude scores, the participants were given a score such that higher scores indicated more positive attitudes. We computed the total scores and percentages for each participant’s knowledge and attitude. The total scores were categorized based on Bloom’s cutoff points for knowledge and attitude: ≤59% for poor knowledge or negative attitude, 60-79% for average knowledge or neutral attitude, and 80-100% for good knowledge or positive attitude.

Ethical considerations

This study was conducted at the Department of Dermatology and approved by the Research Ethics Committee of King Abdulaziz University Hospital. The study was conducted in accordance with the tenets of the Declaration of Helsinki, and all participant records and data generated were managed under strict confidentiality.

Statistical analysis

Descriptive statistics were performed using frequency and percentages, while analytical statistics were performed using the chi-square test to investigate the association and/or difference between two categorical variables, and a p-value < 0.05 was considered statistically significant. Data entry and statistical analyses were performed using SPSS Statistics, version 28 (IBM, Armonk, NY).

## Results

Sociodemographic characteristics of the participants

A total of 526 participants were included in the study. Sociodemographic characteristics of the cohort are presented in Table 1. The majority (78.3%) were male, and 57% were aged ≥40 years. Almost two-thirds (62.4%) of the participants were married, 38.2% were employed, and 80.2% were university graduates. The monthly income of 63.3% was ≤ 5000 Riyals/month (Table 1).

**Table 1:** Sociodemographic characteristics of the participants (n = 526)

Variable	Category	Frequency	Percentage
Gender	Male	114	21.7%
	Female	412	78.3%
Age group (years)	18–20	49	9.3%
	21–39	177	33.7%
	>40	300	57.0%
Marital status	Single	164	31.2%
	Married	328	62.4%
	Divorced	26	4.9%
	Widowed	8	1.5%
Educational level	Below high school	8	1.5%
	High school	96	18.3%
	University/above	422	80.2%
Occupation	Unemployed	219	41.6%
	Employed	201	38.2%
	Student	106	20.2%
Monthly income (Saudi Riyals)	≤ 5000	333	63.3%
	> 5000	193	36.7%

Awareness about psoriasis

**Figure 1:**
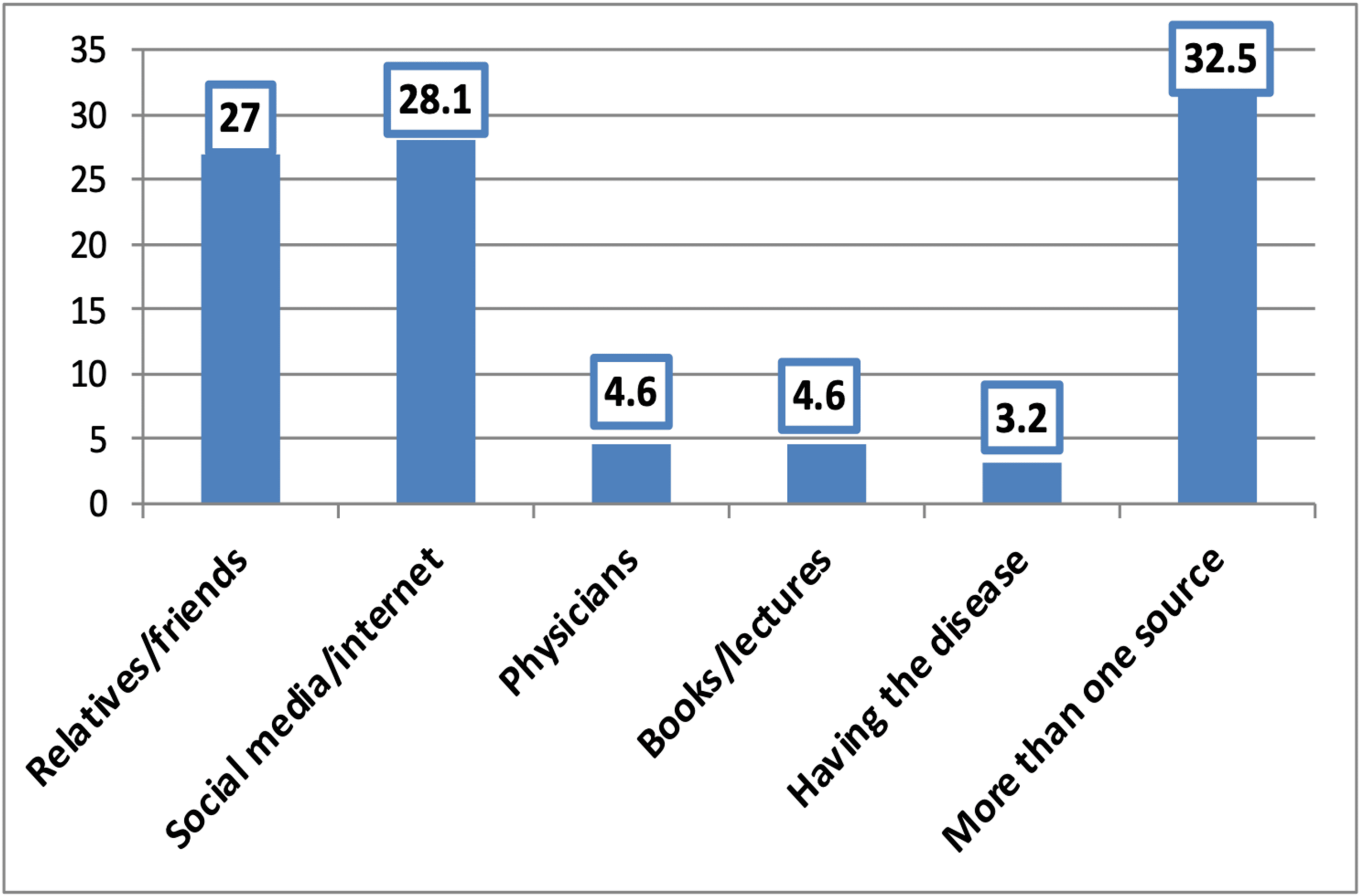
Source of information about psoriasis among the participants

The majority (89.5%) of participants had heard or read about psoriasis, whereas only 17.9% had heard or read about the International Day of Psoriasis. The main source of information about psoriasis was social media/the Internet (28.1%), followed by relatives and friends (27.0%), while more than one source was mentioned by 32.5% of participants (Figure 1).

Knowledge about psoriasis

The majority of the participants (84.4%) correctly recognized that psoriasis is not an infectious disease, while most (74.7%) were aware that patients with eczema are more susceptible to psoriasis. Notably, 58.7% were aware that specific types of food could exacerbate psoriasis (Table 2). Overall, 39.7% of participants demonstrated a good level of knowledge about psoriasis, whereas 20.3% demonstrated a poor level.

**Table 2:** Responses of the participants to knowledge-related questions about psoriasis

Statement/query	Response	Frequency	Percentage
History of hearing/reading about psoriasis disease	No	55	10.5%
	Yes	471	89.5%
History of hearing/reading about the International Day of Psoriasis	No	94	17.9%
	Yes	432	82.1%
Source of information about the psoriasis disease	Relatives/friends	143	27.0%
	Social media/internet	149	28.1%
	Physicians	25	4.6%
	Books/lectures	25	4.6%
	Having the disease	12	2.3%
	More than one source	171	32.5%

Participants with a university education or higher were more likely to demonstrate a good level of knowledge about psoriasis than those with lower levels of education (p = 0.029). Other factors (sex, age, marital status, occupation, monthly income, and source of information) were not significantly associated with knowledge level about psoriasis (Table 3).

**Table 3:** Factors associated with participants' knowledge about psoriasis

Variable	Category	Poor knowledge	Average knowledge	Good knowledge	P-value	Chi-square value (X^2^)
Gender	Male (n = 114)	30 (26.3%)	41 (36.0%)	43 (37.7%)	0.195	3.273
	Female (n = 412)	77 (18.7%)	169 (41.0%)	166 (40.3%)		
Age group (years)	18-20 (n = 49)	9 (18.4%)	22 (44.9%)	18 (36.7%)	0.236	7.194
	21-39 (n = 177)	28 (15.8%)	69 (39.0%)	80 (45.2%)		
	≥40 (n = 300)	70 (23.3%)	119 (39.7%)	111 (37.0%)		
Marital status	Single (n = 164)	29 (17.7%)	65 (39.6%)	70 (42.7%)	0.303	7.194
	Married (n = 328)	70 (21.3%)	128 (39.0%)	130 (39.7%)		
	Divorced (n = 26)	6 (23.1%)	11 (42.3%)	9 (34.6%)		
	Widowed (n = 8)	2 (25.0%)	6 (75.0%)	0 (0.0%)		
Educational level	Below high school (n = 8)	1 (12.5%)	4 (50.0%)	3 (37.5%)	0.029	7.766
	High school (n = 96)	26 (27.1%)	43 (44.8%)	27 (28.1%)		
	University/above (n = 422)	80 (19.0%)	163 (38.6%)	179 (42.4%)		
Occupation	Unemployed (n = 219)	43 (19.6%)	90 (41.1%)	86 (39.3%)	0.982	0.402
	Employed (n = 201)	42 (20.9%)	80 (39.8%)	79 (39.3%)		
	Student (n = 106)	22 (20.8%)	40 (37.7%)	44 (41.5%)		
Monthly income (Saudi Riyals)	≤ 5000 (n = 333)	70 (21.0%)	133 (39.9%)	130 (39.1%)	0.854	0.316
	> 5000 (n = 193)	37 (19.2%)	77 (39.9%)	79 (40.9%)		

Attitudes toward patients with psoriasis

As shown in Table 4, 81% of participants were willing to live in the same household as a patient with psoriasis, 80.2% would be willing to shake hands, and 72.1% would be willing to share food with them, while 52.9% would be willing to use the same swimming pool with a patient, and 51.3% would be willing to marry such a person. Overall, 60.3% of the participants demonstrated a positive attitude toward patients with psoriasis, whereas 27% demonstrated a negative attitude toward them.

**Table 4:** Attitude of the participants towards patients with psoriasis

Attitude and perception-related questions	Frequency	Percentage
Will you share food with the patient with psoriasis?		
No	147	27.9
Yes	379	72.1
Will you shake hands with the patient with psoriasis?		
No	104	19.8
Yes	422	80.2
Will you use the same swimming pool with patients with psoriasis?		
No	248	47.1
Yes	278	52.9
Will you live in the same household with a patient with psoriasis?		
No	100	19
Yes	426	81
Will you enter into a personal relationship (marriage) with the patient having psoriasis?		
No	256	48.7
Yes	270	51.3

The only two factors significantly associated with participants’ attitudes and perceptions toward patients with psoriasis were the source of information and the level of knowledge about the disease. The majority of the participants who obtained their information from books or lectures (83.4%) or who had the disease themselves (82.3%) expressed positive attitudes and perceptions toward patients with psoriasis, compared to only 54.1% of those who obtained their information from social media, the internet, or physicians (p = 0.042). There was also a significant positive association between a higher level of knowledge about the disease and positive attitudes and perceptions toward it (p < 0.001). Other studied factors (participants' sex, age, marital status, occupation, education, and monthly income) were not significantly associated with attitudes and perceptions toward patients with psoriasis, as shown in Table 5.

**Table 5:** Factors associated with participants' attitude towards patients with psoriasis

Variable	Category	Negative attitude, n (%)	Neutral attitude, n (%)	Positive attitude, n (%)	P-value	Chi-square (X²)
Gender	Male (n = 114)	31 (27.2)	17 (14.9)	66 (57.9)	0.712	0.678
	Female (n = 412)	111 (26.9)	50 (12.1)	251 (60.9)		
Age group (years)	18–20 (n = 49)	18 (36.7)	9 (18.4)	22 (44.9)	0.136	6.996
	21–39 (n = 177)	41 (23.2)	20 (11.3)	116 (65.5)		
	≥ 40 (n = 300)	83 (27.7)	38 (12.7)	179 (59.6)		
Marital status	Single (n = 164)	41 (25.0)	23 (14.0)	100 (61.0)	0.325	6.951
	Married (n = 328)	95 (29.0)	37 (11.3)	196 (59.7)		
	Divorced (n = 26)	5 (19.2)	4 (15.4)	17 (65.4)		
	Widowed (n = 8)	1 (12.5)	3 (37.5)	4 (50.0)		
Educational level	Below high school (n = 8)	0 (0.0)	2 (25.0)	6 (75.0)	0.227	5.649
	High school (n = 96)	32 (33.3)	12 (12.5)	52 (54.2)		
	University/above (n = 422)	110 (26.1)	53 (12.6)	259 (61.4)		
Occupation	Unemployed (n = 219)	60 (27.4)	29 (13.2)	130 (59.4)	0.784	1.737
	Employed (n = 201)	52 (25.9)	22 (10.9)	127 (63.2)		
	Student (n = 106)	30 (28.3)	16 (15.1)	60 (56.6)		
Monthly income (Saudi Riyals)	≤ 5000 (n = 333)	85 (25.5)	42 (12.6)	206 (61.9)	0.571	1.122
	> 5000 (n = 193)	57 (29.5)	25 (13.0)	111 (57.5)		
Source of information	Relatives/friends (n = 142)	41 (28.9)	20 (14.1)	81 (57.0)	0.042	118.886
	Social media/internet (n = 148)	48 (32.4)	20 (13.5)	80 (54.1)		
	Physicians (n =2 4)	10 (41.7)	1 (4.2)	13 (54.1)		
	Books/lectures (n = 24)	2 (8.3)	2 (8.3)	20 (83.4)		
	Having the disease (n = 17)	2 (11.8)	1 (5.9)	14 (82.3)		
	More than one source (n = 171)	39 (22.8)	23 (13.5)	109 (63.7)		
Knowledge about psoriasis	Poor (n = 107)	52 (48.6)	11 (10.3)	44 (41.1)	< 0.001	36.055
	Average (n = 210)	54 (25.7)	28 (13.3)	128 (61.0)		
	Good (n = 209)	36 (17.2)	28 (13.4)	145 (69.4)		

## Discussion

National healthcare programs and public initiatives such as World Psoriasis Day have helped increase awareness about psoriasis [16]. Furthermore, in response to the perceived stigma of psoriasis, major steps have been taken to improve public acceptance of the condition and to promote awareness of psoriasis as a noncommunicable chronic inflammatory disorder [14,17]. The prevalence of psoriasis in the general population of Jeddah, Saudi Arabia, remains unknown. Thus, we conducted this survey to assess public perceptions of psoriasis. In our survey, which is the first to assess psoriasis awareness among the general population in the western region of Saudi Arabia, 89.5% of participants reported having heard of the condition. Similarly, prior research found that the majority of participants had read or heard about psoriasis [16-18]. However, familiarity with a disease does not necessarily indicate a thorough understanding of it. For example, social media was the primary source of information for participants, followed by relatives and friends. This may contribute to the spread of misinformation about psoriasis [18]. Furthermore, only 17.9% of participants were aware of the International Day of Psoriasis.

In the current survey, 84.4% of participants correctly understood that psoriasis was not infectious. Similarly, Alraddadi et al. found that 64.0% of their study participants recognized that psoriasis was not infectious [17]. Notably, 20.3% of the participants in the present study believed that psoriasis was infectious, and 20.3% demonstrated limited awareness of the disease. According to Almutairi et al., 12% of study participants believe that psoriasis is communicable [18]. Furthermore, a study conducted in Al-Qassim among Saudi university students found that 18.6% considered psoriasis to be a communicable disease [19]. In our survey, 27.9% of the participants reported that they would not eat at the same table as a patient with psoriasis, 19.8% would not shake hands, 47.1% would not swim in the same pool as a patient with psoriasis, and 48.15% would not form close friendships with a patient with psoriasis. Similarly, Al Mutairi et al. reported that 54% of the participants in their study stated that they would not swim in the same pool as a psoriasis patient, 32% would not form personal relationships with a patient with psoriasis, and 18.2% would not shake hands with a patient with psoriasis [4].

Sommer et al. reported in their study that 7% of respondents were unwilling to eat at the same table as a person with psoriasis, 13% were unwilling to shake hands, 23% were unwilling to share the same swimming pool, and 27% were unwilling to have a personal relationship with a patient with psoriasis [16]. These responses to the stigmatization questions indicate that some members of the general public continue to exhibit prejudice and discriminatory behaviors when interacting with patients with psoriasis, thus preventing them from engaging in important social interactions [20,21]. Furthermore, our study identified a significant association between higher levels of knowledge and more positive attitudes toward patients with psoriasis. Respondents who acquired knowledge about the condition through books, lectures, or personal experience had considerably higher levels of knowledge and more positive attitudes toward patients with psoriasis. This highlights the need for enhanced public awareness efforts.

## Conclusions

This study found that only 39.7% of the participants demonstrated a good level of knowledge, and 60.3% demonstrated a positive attitude toward psoriasis. Higher education was significantly associated with better knowledge, while positive attitudes were significantly linked to obtaining information through books or lectures, personal experience with the disease, and higher levels of knowledge. Awareness of psoriasis needs to be improved among the general population, and information should be effectively disseminated through medical conferences, articles, social media posts, and symposia. In addition, we recommend that healthcare practitioners develop and implement initiatives that enhance public education and awareness campaigns to dispel misconceptions about psoriasis and its impact. The findings of this study should be validated in larger-scale studies with more representative samples, including nationwide populations.

## References

[1] Augustin M, Glaeske G, Radtke MA, Christophers E, Reich K, Schäfer I (2010). Epidemiology and comorbidity of psoriasis in children. Br J Dermatol.

[2] Rendon A, Schäkel K (2019). Psoriasis pathogenesis and treatment. Int J Mol Sci.

[3] Trembath RC, Clough RL, Rosbotham JL (1997). Identification of a major susceptibility locus on chromosome 6p and evidence for further disease loci revealed by a two stage genome-wide search in psoriasis. Hum Mol Genet.

[4] Nair RP, Stuart PE, Nistor I (2006). Sequence and haplotype analysis supports HLA-C as the psoriasis susceptibility 1 gene. Am J Hum Genet.

[5] Armstrong AW, Read C (2020). Pathophysiology, clinical presentation, and treatment of psoriasis: a review. JAMA.

[6] Christophers E (2001). Psoriasis--epidemiology and clinical spectrum. Clin Exp Dermatol.

[7] Parisi R, Symmons DPM, Griffiths CEM, Ashcroft DM (2013). Identification and Management of Psoriasis and Associated ComorbidiTy (IMPACT) project team. Global epidemiology of psoriasis: a systematic review of incidence and prevalence. J Invest Dermatol.

[8] Fatani MI, Abdulghani MH, Al-Afif KA (2002). Psoriasis in Eastern Saudi Arabia. Saudi Med.

[9] Alzeer F, AlOtair H, Aleisa A (2022). Epidemiology and cutaneous manifestations of psoriasis in Saudi Arabia: a narrative review. Clin Cosmet Investig Dermatol.

[10] Korman NJ (2020). Management of psoriasis as a systemic disease: what is the evidence?. Br J Dermatol.

[11] Michalek IM, Loring B, John SM (2017). A systematic review of worldwide epidemiology of psoriasis. J Eur Acad Dermatol Venereol.

[12] Dand N, Mahil SK, Capon F, Smith CH, Simpson MA, Barker JN (2020). Psoriasis and genetics. Acta Derm Venereol.

[13] Feldman SR, Malakouti M, Koo JY (2014). Social impact of the burden of psoriasis: effects on patients and practice. Dermatol Online J.

[14] (2026). World Health Organization. Global report on psoriasis. https://iris.who.int/handle/10665/204417.

[15] Hrehorów E, Salomon J, Matusiak L, Reich A, Szepietowski JC (2012). Patients with psoriasis feel stigmatized. Acta Derm Venereol.

[16] Sommer R, Mrowietz U, Radtke MA (2018). What is psoriasis? Perception and assessment of psoriasis among the German population. J Dtsch Dermatol Ges.

[17] Olaythah Alraddadi M, Ibrahim Alhawiti SA, Bakheet Alotaibi AN (2020). Knowledge, perception, and attitude about psoriasis among the general population in Tabuk, Saudi Arabia. Saudi Med J Stud.

[18] Almutairi S, Alotaibi A, Almohideb M (2020). Perception and assessment of psoriasis in the general population of Riyadh, Saudi Arabia. Saudi J Health Sci.

[19] Parisi R, Webb RT, Kleyn CE, Carr MJ, Kapur N, Griffiths CEM, Ashcroft DM (2019). Psychiatric morbidity and suicidal behaviour in psoriasis: a primary care cohort study. Br J Dermatol.

[20] Weiss SC, Kimball AB, Liewehr DJ, Blauvelt A, Turner ML, Emanuel EJ (2002). Quantifying the harmful effect of psoriasis on health-related quality of life. J Am Acad Dermatol.

[21] Chaturvedi SK, Singh G, Gupta N (2005). Stigma experience in skin disorders: an Indian perspective. Dermatol Clin.

